# Study filters for non-randomized studies of interventions consistently lacked sensitivity upon external validation

**DOI:** 10.1186/s12874-018-0625-4

**Published:** 2018-12-18

**Authors:** Elke Hausner, Maria-Inti Metzendorf, Bernd Richter, Fabian Lotz, Siw Waffenschmidt

**Affiliations:** 1 Information Management Unit, Institute for Quality and Efficiency in Health Care (IQWiG), Im Mediapark 8, 50670 Cologne, Germany; 2 Cochrane Metabolic and Endocrine Disorders Group, Institute of General Practice, Medical Faculty of the Heinrich-Heine-University, Düsseldorf, Germany; 3 Department of Medical Biometry, Institute for Quality and Efficiency in Health Care (IQWiG), Im Mediapark 8, 50670 Cologne, Germany

**Keywords:** Information storage and retrieval, Databases, bibliographic, Medline, Sensitivity and specificity, Reproducibility of results

## Abstract

**Background:**

Little evidence is available on searches for non-randomized studies (NRS) in bibliographic databases within the framework of systematic reviews. For instance, it is currently unclear whether, when searching for NRS, effective restriction of the search strategy to certain study types is possible. The following challenges need to be considered: 1) For non-randomized controlled trials (NRCTs): whether they can be identified by established filters for randomized controlled trials (RCTs). 2) For other NRS types (such as cohort studies): whether study filters exist for each study type and, if so, which performance measures they have.

The aims of the present analysis were to identify and validate existing NRS filters in MEDLINE as well as to evaluate established RCT filters using a set of MEDLINE citations.

**Methods:**

Our analysis is a retrospective analysis of study filters based on MEDLINE citations of NRS from Cochrane reviews. In a first step we identified existing NRS filters. For the generation of the reference set, we screened Cochrane reviews evaluating NRS, which covered a broad range of study types. The citations of the studies included in the Cochrane reviews were identified via the reviews’ bibliographies and the corresponding PubMed identification numbers (PMIDs) were extracted from PubMed. Random samples comprising up to 200 citations (i.e. 200 PMIDs) each were created for each study type to generate the test sets.

**Results:**

A total of 271 Cochrane reviews from 41 different Cochrane groups were eligible for data extraction. We identified 14 NRS filters published since 2001. The study filters generated between 660,000 and 9.5 million hits in MEDLINE. Most filters covered several study types. The reference set included 2890 publications classified as NRS for the generation of the test sets. Twelve test sets were generated (one for each study type), of which 8 included 200 citations each. None of the study filters achieved sufficient sensitivity (≥ 92%) for all of the study types targeted.

**Conclusions:**

The performance of current NRS filters is insufficient for effective use in daily practice. It is therefore necessary to develop new strategies (e.g. new NRS filters in combination with other search techniques). The challenges related to NRS should be taken into account.

**Electronic supplementary material:**

The online version of this article (10.1186/s12874-018-0625-4) contains supplementary material, which is available to authorized users.

## Background

Randomized controlled trials (RCTs) show the highest certainty of results of all study types, provided their methods were correct and implemented in a way suitable to address a study’s objectives. For the assessment of the benefit of medical interventions within the framework of systematic reviews, well conducted RCTs thus provide results with the lowest risk of bias.

The inclusion of non-randomized studies (NRS) in the assessment of interventions leads to a markedly higher risk of bias [1]. There are, however, cases in which the evidence from RCTs is insufficient to be able to assess the patient-relevant benefit and harm of an intervention, so that NRS are also used.

It is currently unclear whether, when searching for NRS in bibliographic databases, effective restriction of the search to certain study types is possible. Methodological study filters are usually used for this purpose.

Jenkins [2] describes 3 different types of study filters in her review: subjectively derived without calculation of performance measures (first generation), subjectively derived and tested against a set of independent citations, i.e. a known set of relevant citations (second generation), as well as objectively derived and tested against a set of independent citations (third generation). Well-established third-generation search filters are currently available with the Cochrane Highly Sensitive Search Strategy Filters [3] and the search filters of the Health Information Research Unit (HIRU) of McMaster University [4].

NRS include all study types except RCT. When searching for NRS it needs to be considered that NRS comprise different study types (see items 2 to 12 in Table 2). These also include non-randomized controlled trials (NRCTs), i.e. trials where randomization cannot be excluded or was inadequate [3]. NRCTs are of particular relevance as they are often considered in systematic reviews in addition to RCTs. In the present article we use NRS as an umbrella term for non-randomized studies and NRCT as a specific study type within NRS.

The different NRS types are not consistently labelled in the literature [5]*.* This is also why precise information on the study type is often lacking in the titles and abstracts of publications. It is unclear whether indexing in bibliographic databases such as MEDLINE can compensate this deficit.

It is therefore necessary to analyse the search for NRS in bibliographic databases and develop an adequate approach for identifying these studies. The following challenges exist:

1. For NRCTs, the question arises as to whether they can be identified by established RCT filters [3, 4]. For instance, Glanville et al. [6] report that they developed the Cochrane Highly Sensitive Search Strategy Filter by means of RCTs. However, both RCTs and NRCTs were used to measure its performance.
2. It is unclear whether study filters exist for other NRS study types (such as cohort, case-control or cross-sectional studies), and if so, how they were developed (approach for first- to third-generation filters) and how they perform

### Objectives

The aims of the present analysis were

- to identify and validate existing NRS filters in MEDLINE
- to evaluate established RCT filters with regard to whether they can also identify NRCTs using a set of MEDLINE citations.

## Methods

The present analysis is a retrospective analysis of study filters by means of MEDLINE citations on NRS from Cochrane reviews. As MEDLINE is the most frequently used bibliographic database in medicine [7], our analysis was restricted to this source.

### Approach applied

**Fig. 1:**
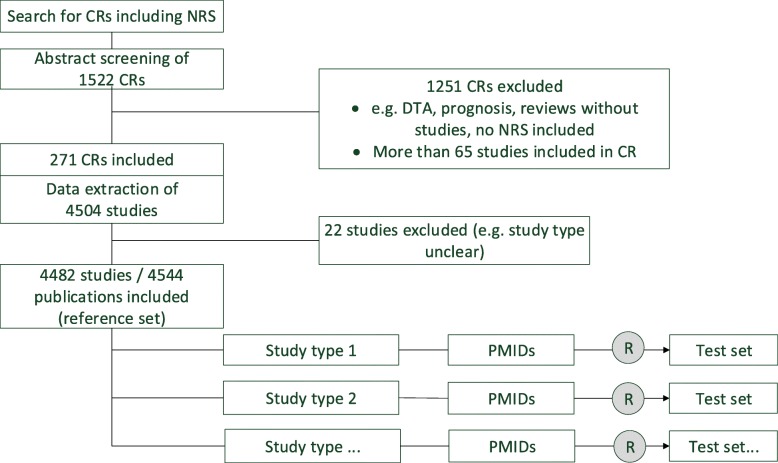
Flowchart for generation of the test sets

We generated test sets to address the study’s objectives. The following section describes our approach; the different working steps are shown in the flowchart in Fig. 1.

### Identification of Cochrane reviews

We analysed Cochrane reviews to generate the reference set, as they represent high-quality systematic reviews following international evidence-based standards and, due to a standard template, contain a more consistent presentation of the study types included compared with non-Cochrane reviews. Most Cochrane reviews are based on RCTs, but some also consider NRS or are based exclusively on them.

To identify Cochrane reviews, we modified the search syntax by Ijaz et al. [8] (see Additional file 1). Like Ijaz et al., we excluded search terms for quasi-randomized or controlled clinical trials. A search for these specific NRS types is not meaningful, as searches conducted in most Cochrane reviews based on RCTs also target these two study types: searching for them would thus make it more difficult to identify Cochrane reviews largely including NRS.

Our analysis considered all Cochrane reviews corresponding to the pre-specified inclusion criteria (see Table 1) and identified by means of the screening of abstracts. For example, the Cochrane reviews had to evaluate an intervention on a health-related question and had to include study types beyond RCTs or NRCT.

**Table 1:** Inclusion criteria for Cochrane reviews (after abstract screening)

Inclusion criteria	
I1a	Most current version of a Cochrane review
I2a	A Cochrane review evaluating an intervention on a health-related question (e.g. can also include topics from the field of public health)
I3a	A Cochrane review not only including RCTs or NRCT
I4a	A Cochrane review including NRS (based on the section “Main results” of the abstract of the Cochrane review)
I5a	A Cochrane review including < 65 studies

We specified a priori that a total of 4500 study publications would be required. In order to avoid the domination of individual Cochrane reviews, in a randomized sequence we first extracted all Cochrane reviews containing fewer than 50 studies, and in a second step extracted those reviews containing fewer than 65 studies. Ten Cochrane reviews containing more than 65 studies were excluded.

All eligible Cochrane reviews published up to 20 October 2016 were considered.

To document their wide range of topics, the Cochrane reviews were classified according to the intervention type and level, following Polus et al. [9] (see Additional file 2).

### Generation of the reference set

One person extracted the studies included in the eligible Cochrane reviews, together with the information on the study type, for the generation of a reference set. As a quality assurance step, data extraction was checked by a second person for 5% of the Cochrane reviews. We primarily extracted the information on the study type (see Table 2) from the “Characteristics of included studies” tables, the “Additional tables”, the “Appendices” and, if appropriate, the “Results” section of the Cochrane review. As RCTs were also included in some of the eligible Cochrane reviews, for reasons of completeness they were also extracted.

**Table 2:** Study types extracted

Coding	Terms (following Hartling et al. [10])
1	Randomized controlled trial (RCT)
2	Non-randomized controlled trial (NRCT)^a)^
3	Controlled before-after study
4	Interrupted time series (with comparison group)
5	Prospective cohort study
6	Retrospective cohort study
7	Non-concurrent cohort study
8	(Nested) case-control study
9	Cross-sectional study
10	Non-comparative study (e.g. case report or case series)
11	Before-after study
12	Interrupted time series (without comparison group)

^a)^Also refers to quasi-randomized controlled trial and controlled clinical trial

For 606 studies, a clear allocation of study type was not possible on the basis of the information provided in the Cochrane reviews. We performed a post-hoc classification of these studies on the basis of the abstracts, following the classification scheme by Hartling et al. [10]. Even after this step, 23% of the 606 studies could not be clearly allocated to a study type.

The citations of the primary studies included were identified via the “References to studies included in this review” section, and the corresponding PubMed identification numbers (PMIDs) were extracted from PubMed.

### Generation of the test sets

The test sets for the evaluation of NRS filters contained those citations that could be allocated to a study type (see Table 2). Citations without a PubMed entry were counted and documented, but not included in the test sets. After determining the citations to be included, as well as the corresponding study types, random samples comprising 200 citations each were created for each study type to generate the test sets (see section on sample size calculation). An overview of the methods for generating the test sets is presented in Fig. 1.

### Statistical analyses

#### Sample size calculation

We planned to evaluate existing study filters with regard to sensitivity and specificity (see Additional file 2), and aimed to identify 200 PMIDs per study type in order to obtain reliable conclusions on sensitivity. Depending on the topic investigated, sensitivities between 90 and 98% are required for the generation of systematic reviews [6, 11–15]. To achieve a sufficient performance, we specified a sensitivity for the study filter of at least 95%. Following Sampson’s sample size calculation [16], we determined an interval within which the sensitivity measured must lie in order to cover the actual sensitivity of at least 95%.

For a sample of 200 PMIDs per study type, if the filter’s sensitivity lies within the interval of [0.92;1], it cannot be excluded that the actual sensitivity is 95%. If sensitivity is < 91% for the same sample size, it is highly likely that the filter has an actual sensitivity of less than 95%. Because of this estimation, at least 200 PMIDs per study type should be used for an evaluation of sensitivity.

If fewer than 200 PMIDs were available for certain study types, this was described in the results section and it was estimated how this smaller number affected the evaluation of sensitivity. If the number of PMIDs was higher for a study type, then a random sample of 200 was drawn (see generation of the test set) from all available PMIDs for this study type (reference set). A similar approach would have been difficult to implement for specificity, as the number of wrongly identified studies cannot be reliably estimated and specificity may possibly be very low. However, the sensitivity of a filter is the more important performance measure, which is why the calculation of the test set on the basis of sensitivity seemed sufficient.

### Study filters

#### Identification of existing filters

The following sources were searched to identify NRS filters: the website of the InterTASC Information Specialists’ Sub-Group [17], IQWiG’s internal literature collection on information retrieval, as well as MEDLINE following the approach by Belisario et al. [18]. The search filters from all 3 sources were documented and information on them extracted (see Additional file 3).

Search filters were considered that had been developed for the MEDLINE search interfaces PubMed or Ovid SP and published from 2001 onwards. If a study filter was available for both interfaces, only Ovid SP was tested. The established RCT filters by Cochrane and HIRU [3, 4] were used to evaluate RCTs and NRCTs.

#### Evaluation of existing study filters

We entered the study filters and PMIDs identified into MEDLINE (Ovid SP). We linked the search results of the study filters with the PMIDs of the respective test sets by means of the AND operator and calculated sensitivity.

We regarded study filters with a sensitivity of ≥92% to be sufficiently sensitive for the present analysis. If a study filter reached a sensitivity of ≥92%, we planned to calculate its specificity.

## Results

### Reference set

We initially identified 1522 Cochrane reviews in PubMed. After the screening of abstracts by 2 reviewers independently of one another, 271 eligible Cochrane reviews remained for data extraction. Of these, 140 (52%) used an NRS filter in their search strategies; for 9 (3%) it was unclear whether this type of filter was used or not. The information extracted from the Cochrane reviews yielded 4482 studies for the reference set. These corresponded to 5815 documents of which 4544 were available in MEDLINE; 2890 studies were classified as NRS (see Table 3). No Pubmed entry was identified for 631 studies (14%) from the reference set.

**Table 3:** Characteristics of the reference set

Study type	Number of CRs^a)^	Number of studies	Number of PMIDs^b)^
Randomized controlled trial	183	1471	1598
Nonrandomized controlled trial	67	216	331
Controlled before-after study	104	634	556
Interrupted time series (with comparison group)	31	83	106
Prospective cohort study	84	384	435
Retrospective cohort study	72	436	451
Non-concurrent cohort study	13	34	31
(Nested) case-control study	36	207	200
Cross-sectional study	17	152	136
Non-comparative study (case report or case series)	22	249	226
Before-after study	41	257	239
Interrupted time series (without comparison group)	45	221	179
Study type unclear	42	138	56
**Total**	**271**	**4482**	4544 **(2890 on NRS)**

^a^)Number of CRs in which the study type was included at least once (multiple counting possible)

^b^)Without duplicates

**Fig. 2:**
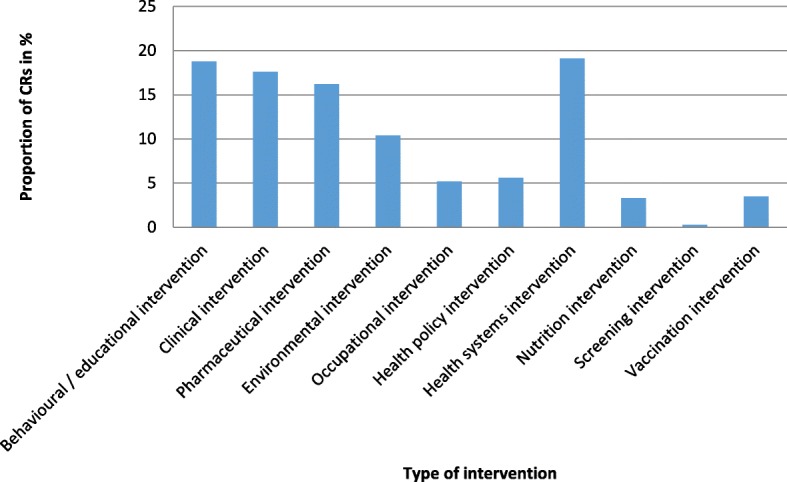
Type of intervention examined by Cochrane reviews in the reference set (according to Polus et al. [9])

The 271 extracted Cochrane reviews originated from 41 different Cochrane groups (see Additional file 4) and covered a wide range of topics (see Fig. 2); 6 groups generated more than half of the reviews included. The “Effective Practice and Organization of Care” group generated the highest number of reviews, as this group examines topics often not investigated with RCTs.

Figure 2 shows the types of interventions of the studies considered in the Cochrane reviews and included in the reference set. The 4 most common interventions representing three-quarters of the interventions in the reference set were health systems, behavioural/educational, clinical, and pharmaceutical intervention.

### Overview of the study filters

A total of 14 NRS filters published since 2001 by 6 different filter developers were identified: 9 filters were classified as first-generation and 5 as third-generation filters. The latter achieved sensitivities between 68.6 and 99.5%. The 14 NRS filters generated between 660.000 and 9.5 million hits in MEDLINE (Ovid SP).

Details on the study filters such as source, complete syntax, filter generation as well as performance measures, if available, are presented in Table 4 and in detail in Additional file 3.

**Table 4:** Overview and evaluation of the NRS filters identified

Study filters (developers)	Study types targeted	Coding^a)^	Hits in MEDLINE	Sensitivity (interval)^b)^
Clinical trials (University of Texas) [27]	Clinical trials	2	1.445.276	39%
Observational Studies – Medline (SIGN) [20]	Observational studies	5–10	2.492.125	49–90%
MEDLINE precision (Fraser 2000) [24]	Observational studies	5–8,10	9.509.757	73–88%
MEDLINE specificity (Fraser 2000) [24]	Observational studies	5–8,10	8.423.107	53–85%
MEDLINE cohort, case-control, and case series strategy (BMJ) [28]	Observational studies	5–8,10	2.517.309	55–92%
MEDLINE cohort, case-control, case series, and case study strategy (BMJ) [28]	Observational studies	5–8,10	4.441.461	61–93%
Search terms for finding non-RCTs (Royle 2003) [29]	Non-RCT	2–12	8.073.091	46–98%
MEDLINE cohort study strategy (BMJ) [28]	Cohort studies	5–7	1.982.782	58–69%
Cohort studies (University of Texas) [27]	Cohort studies	5–7	2.204.911	52–72%
Case-control studies_1 (University of Texas) [27]	Case-control studies	8	660.864	78%
Case-control studies_2 (University of Texas) [27]	Case-control studies	8	1.284.387	80%
Medline cohort and case-control strategy (BMJ) [28]	Cohort, case-control	5–8	2.430.887	61–92%
Fixed method A for MEDLINE (Furlan 2006) [21]	Cohort, case-control, cross-sectional	5–9	4.184.894	49–83%
Fixed method B for MEDLINE (Furlan 2006) [21]	Cohort, case-control, cross-sectional	5–9	6.559.073	69–85%

^a^) Coding of study types; see Table 2; ^b^) Presentation as an interval if the study filter covers more than one study type

*SIGN* Scottish Intercollegiate Guidelines Network, *BMJ* British Medical Journal Clinical Evidence

### Results of the evaluation of the NRS filters

A total of 2890 classified publications on NRS were available in the reference set for the generation of the test sets (see Table 3). Seven complete test sets per study type (i.e. with 200 citations each) could be generated. The test sets for 4 study types contained fewer citations: interrupted time series (with comparison group), non-concurrent cohort study, cross-sectional study, and interrupted time series (without comparison group), (see Table 3).

Table 4 shows an overview of the NRS filters identified, the study types targeted, and the results of the filter evaluation on the basis of sensitivity. “Study types targeted” refers to those study types that were to be identified by the filters according to the filter developers or filter names, or were presumably to be identified by the filter by means of the search terms listed. Most filters covered several study types, which is why their sensitivity is presented as an interval within which the different sensitivities for the different study types targeted are shown. The details of the filter testing are presented in Additional file 5.

The sensitivities presented in Table 4 show that although some filters achieved sufficient sensivity (see the section “Evaluation of existing study filters”) for individual study types, none achieved sufficient sensitivity for all of the study types targeted. Therefore the overall performance of the filters is insufficient to effectively apply them in practice (see Additional file 5). As this applied to all 14 filters evaluated, we did not calculate specificity.

### Results of the evaluation of RCT filters for NRCTs

We also evaluated whether established RCT filters are suited to reliably identify NRCTs and how they perform in an independent setting (see Table 5).

**Table 5:** Evaluation of established RCT filters

Study filters	Hits in MEDLINE	Sensitivity NRCTs
Therapy Medline (Haynes 2005) – max. Sensitivity	5.213.988	54%
Therapy Medline (Haynes 2005) – max. Specifity	485.918	13%
Therapy Medline (Haynes 2005) – optimizing sensitivity/specifity	796.127	16%
Cochrane Search Strategy (2008) – sensitivity-max.	3.581.596	57%
Cochrane Search Strategy (2008) – sensitivity and precision-max.	1.057.717	37%

Table 5 shows that the maximized sensitivity filters by Haynes (HIRU McMaster University) and Cochrane did not yield sufficient sensitivity to identify NRCTs.

### Post-hoc analyses

#### Validation of NRS filters by means of specific intervention types

As overall, the performance of the filters was insufficient, we conducted post-hoc subgroup analyses on the basis of specific intervention types. Our hypothesis was that filters applied for research questions excluding the area “Public health and health systems” performed better than filters including this area. We therefore conducted a subgroup analysis for the following intervention types:

- Behavioral/education intervention
- Clinical intervention
- Pharmaceutical intervention
- Nutrition intervention
- Screening intervention

However, for all study types targeted, sensitivity only increased slightly and still lay consistently below 92%. Additional file 5 contains detailed results on the filter evaluation in this subgroup compared with the validation referring to the whole reference set.

#### Validation of RCT and NRS filters by means of publications allocated to studies

In addition, the data analysis showed that the reference set also contained publications that should not have been allocated to the study type extracted. This particularly refers to cases where 2 or more publications were available for the same study: even if the primary publication was correctly allocated to a study type, further publications might represent other study types such as secondary analyses.

In a separate step we therefore tested which study types in the reference set include more than 30% of study citations to which more than one publication was allocated. This was the case for study types 1 to 5 (see Table 2). A subgroup analysis for these filters showed that sensitivity increased between 5 to 15 percentage points for NRCTs, whereas only minor changes were shown for the other study types (see Additional file 6).

## Discussion

In our retrospective analysis of study filters based on MEDLINE citations of NRS from Cochrane reviews, no known NRS filter achieved sufficient sensitivity (≥ 92%), a precondition for comprehensive information retrieval (due to the insufficient sensitivity, we did not evaluate specificity). The question of how to search for NRS thus remains open.

A recent publication by Glanville et al. [19] draws similar conclusions and notes that the identification of NRS should focus on the topic investigated rather than on a specific study design. The authors’ suggestions for solving this problem include better indexing in databases and reporting guidance.

However, the call to dispense with study filters in searches for NRS does not seem to correspond with the usual practice and demand for these search filters. In the present analysis, more than half of Cochrane reviews had a search block for NRS. Filters are being used whose performance was previously unclear and has now been shown to be insufficient by the present analysis. We therefore believe that at least an attempt should be made to develop adeqate NRS filters; the reference set of the present analysis could be used for this purpose.

Due to the broad range of topics and the time period covered, as well as its size, this reference set is unique in the field of NRS. The size and representativeness of a reference set are particularly important to be able to make reliable statements on the performance of study filters [2]. Our reference set is based on a systematic analysis of most available Cochrane reviews considering NRS and, according to the sample size calculation, a sufficient number of publications could be identified for 8 (out of 12) study types with 200 citations each.

The result of the subgroup analysis based on specific intervention types is interesting insofar as our current assumption that the low sensitivity of NRS filters was largely caused by studies from the field of public health and health systems was not confirmed. The sensitivities of the test sets including versus those of the test sets excluding this field only showed minor differences (< 5 percentage points). This did not apply to the cross-sectional studies, where performance increased by 9 to 10% for 3 study filters [20, 21].

For 9 of the 14 filters tested, neither information on filter development nor performance measures were provided, meaning that these filters do not meet current standards [2, 22, 23]. This could have been neglected if the sensitivity of the study filters had been sufficently high in the present analysis. We could not even reproduce the performance of the 2 filters showing sufficient sensitivity (≥ 91%) in [24]. This indicates how important it is to validate study filters with reference sets outside the context of filter development [25].

The evaluation of established RCT filters showed that 2 filters, Therapy Medline (Haynes 2005) – max. Sensitivity [4] and Cochrane Search Strategy (2008) sensitivity-max [3] did not yield sufficient sensitivity to identify NRCTs. The limitations concerning the identification of NRS mentioned above thus also apply to NRCTs. This is of particular interest for those authors of systematic reviews who, besides RCTs, also consider non-randomized study types. In this context it is not only important how the study types are labelled, but also how they are defined. For instance, the Cochrane Handbook defines controlled clinical trials (CCTs) as studies where randomization cannot be excluded or was inadequate [3]. In contrast, the definition by the US National Library of Medicine is far less restrictive and also covers study types such as historical comparisons [26]. But study filters can only be developed in a reliable manner if a generally accepted definition of the study type that is to be identified exists. This problem has also been addressed by Polus et al. [9] for controlled before-after and interrupted time series studies.

In addition, 14% of the studies from the reference set did not have a MEDLINE entry. These included non-MEDLINE indexed journal publications, research reports or other unpublished data. The document type could not be inferred from the data extracted. It thus remains unclear which information sources (e.g. additional bibliographic databases, trial registries) are particularly suited to identify non-MEDLINE-indexed NRS.

### Limitations

The present analysis has the following limitations:

1. The target number of 200 PMIDs per study type could not be reached for 4 study types (interrupted time series with or without a control group, non-concurrent cohort study, and cross-sectional study). The corresponding results thus have limited informative value.
2. No generally accepted classification scheme for NRS currently exists. During data extraction it became clear that even Cochrane authors had difficulties in clearly allocating studies to a certain study type, even though the full publication was available. In the present analysis 606 studies (approx. 14%) that initally could not be clearly allocated to a study type were subsequently classified on the basis of the abstract. Furthermore, only one person allocated these studies to a study type, which could have potentially resulted in misclassifications.
3. We used Cochrane reviews as the basis of our reference set. These reviews have a focus on RCTs and are often conducted within topic groups and/or several searches within the framework of a series of reviews are conducted by the same team. In addition, it is a potential weakness of the relative recall approach to rely on reviews that are only as good as the searches that were conducted to create them.

### Implications for research

The following points should be considered in the development of NRS filters:

1. Representativeness of the reference set: For some study types, multiple publications on the same study were available. These included, for example, secondary publications that did not always match the study type of the primary publication. In order not to jeopardize the representativeness of the test set, only those citations should be used in filter development that are clearly labelled as the primary publication. This accepts that the study filter does not identify each publication on a study. Study filters should thus reliably identify the primary publication; all further publications related to a study can be identified in a separate search step. Moreover, an additional independent test set should be generated for study types with fewer than 200 PMIDs.
2. Study filters across study types: as noted in the limitations, classifying the different study types is a challenging task. It is therefore understandable that the inconsistent definition and labelling of study types prevents the conduct of standardized searches in practice. For the future development of study filters, it should therefore be evaluated whether, compared with existing study filters, broader study types (e.g. controlled versus non-controlled studies) can achieve better performance measures.
3. Addition of further search techniques: a further approach could be to apply study filters in combination with other search techniques (such as the “similar articles” function in Pubmed), thus enabling the use of study filters with a lower sensitivity (e.g. 90%)
4. Filter validation: Filters should be validated using an independent set of references (e.g. extracted from non-Cochrane systematic reviews identified in Epistemonikos or the Campbell Library)

In addition, we recommend clear and mandatory labelling of the study type by authors of primary publications: Editors of scientific journals should demand a clear label for a study type at the time of manuscript submission and this information should be a mandatory part of the structured abstract. In this context, the labelling of study type should not be freely chosen, but chosen from an internationally consented classification scheme. In addition, editors and peer reviewers should check that the study type reported is consistent with the information provided in the methods section of the manuscript.

## Conclusions

The performance of current NRS filters is insufficient for effective use in daily practice. It is therefore necessary to develop new strategies (e.g. new NRS filters in combination with other search techniques). The challenges related to NRS should be taken into account.

## Additional files

Additional file 1: PubMed search strategy for identifying Cochrane reviews with NRS. This file includes the search strategy we used to identify Cochrane reviews with NRS. (PDF 98 kb)

Additional file 2: Classification of the intervention investigated in the Cochrane review; Performance measures. This file includes information on how we classified Cochrane reviews according to the intervention type and level and how we calculated sensitivity and specificity. (PDF 101 kb)

Additional file 3: Overview of study filters. This file includes a documentation of the search filters we identified and information on these filters (PDF 143 kb)

Additional file 4: Number of reviews and studies per Cochrane group in the reference set. This file includes information on the allocation of Cochrane reviews to the Cochrane groups (PDF 83 kb)

Additional file 5: Detailed results of the evaluation of study filters. This file includes details of the filter testing (PDF 356 kb)

Additional file 6: Detailed results of the evaluation of study filters: only one PMID per study. This file includes a subgroup analysis of studies with only one PMID per study. (PDF 119 kb)

## Abbreviations

CCT: Controlled clinical trials
CRs: Cochrane reviews
DTA: Diagnostic test accuracy
HIRU: Health Information Research Unit
IQWiG: Institute for Quality and Efficiency in Health Care
NRCT: Non-randomized controlled trials
NRS: Non-randomized studies
PMID: PubMed identification numbers
PMIDs: PubMed identification numbers
R: Random selection
RCT: Randomized controlled trials
