# Supplementary material for: Study filters for non-randomized studies of interventions consistently lacked sensitivity upon external validation
Source: BMC Med Res Methodol. 2018 Dec 18;18:171. doi: 10.1186/s12874-018-0625-4 (PMC6299552; doi:10.1186/s12874-018-0625-4)
Supplement: Supplementary file 1 — PubMed search strategy for identifying Cochrane reviews with NRS. This file includes the search strategy we used to identify Cochrane reviews with NRS. (PDF 98 kb) [file 12874_2018_625_MOESM1_ESM.pdf]

PubMed search strategy for identifying Cochrane reviews with NRS

Search	Query	Hits
#1	(non random*[tiab] OR nonrandom*[tiab] OR NRS[tiab] OR NRSs[tiab]) AND Cochrane Database Syst Rev[ta]	481
#2	follow up stud*[tiab] AND Cochrane Database Syst Rev[ta]	31
#3	epidemiolog*[tiab] AND Cochrane Database Syst Rev[ta]	94
#4	observational[tiab] AND Cochrane Database Syst Rev[ta]	404
#5	ecologic*[tiab] AND Cochrane Database Syst Rev[ta]	7
#6	longitudinal[tiab] AND Cochrane Database Syst Rev[ta]	21
#7	("case series"[tiab] OR case report*[tiab]) AND Cochrane Database Syst Rev[ta]	169
#8	("interrupted time series"[tiab] OR "interrupted times series"[tiab]) AND Cochrane Database Syst Rev[ta]	257
#9	("before after"[tiab] OR "before and after"[tiab] OR "CBA"[tiab] OR "CBAs"[tiab]) AND Cochrane Database Syst Rev[ta]	347
#10	cross sectional*[tiab] AND Cochrane Database Syst Rev[ta]	45
#11	case control*[tiab] AND Cochrane Database Syst Rev[ta]	132
#12	cohort[tiab] AND Cochrane Database Syst Rev[ta]	280
#13	**#1 OR #2 OR #3 OR #4 OR #5 OR #6 OR #7 OR #8 OR #9 OR #10 OR #11 OR #12**	**1522**

The search was conducted in PubMed on 20.10.2016
