# Supplementary material for: Study filters for non-randomized studies of interventions consistently lacked sensitivity upon external validation
Source: BMC Med Res Methodol. 2018 Dec 18;18:171. doi: 10.1186/s12874-018-0625-4 (PMC6299552; doi:10.1186/s12874-018-0625-4)
Supplement: Supplementary file 2 — Classification of the intervention investigated in the Cochrane review; Performance measures. This file includes information on how we classified Cochrane reviews according to the intervention type and level and how we calculated sensitivity and specificity. (PDF 101 kb) [file 12874_2018_625_MOESM2_ESM.pdf]

Classification of the intervention investigated in the Cochrane review, following Polus et al. [1]

**Type of intervention**
Behavioural / educational intervention
Clinical intervention
Pharmaceutical intervention
Environmental intervention
Occupational intervention
Health policy intervention
Health systems intervention
Nutrition intervention
Screening intervention
Vaccination intervention

**Level of intervention**
Individual
Organisational/group
Large scale/population

Performance measures (following Jenkins [2])

		Relevant (test set)	Not relevant
Search filter	Retrieved	a	b
	Not retrieved	c	d
		a + c	b + d

Sensitivity = a/(a+c); Specificity = d/(b+d)

Reference list

1. Polus S, Pieper D, Burns J, Fretheim A, Ramsay C, Higgins JPT, Mathes T, Pfadenhauer LM, Rehfuess EA: **Heterogeneity in application, design, and analysis characteristics was found for controlled before-after and interrupted time series studies included in Cochrane reviews**. *J Clin Epidemiol* 2017, **91**:56-69.
2. Jenkins M: **Evaluation of methodological search filters: a review**. *Health Info Libr J* 2004, **21**(3):148-163.
