# Supplementary material for: Study filters for non-randomized studies of interventions consistently lacked sensitivity upon external validation
Source: BMC Med Res Methodol. 2018 Dec 18;18:171. doi: 10.1186/s12874-018-0625-4 (PMC6299552; doi:10.1186/s12874-018-0625-4)
Supplement: Supplementary file 3 — Overview of study filters. This file includes a documentation of the search filters we identified and information on these filters (PDF 143 kb) [file 12874_2018_625_MOESM3_ESM.pdf]

Overview of study filters

Study filters (developer)	Source	Syntax according to documentation	Gene-ration	Sensi-tivity	Speci -ficity	Prec-ision
Case-control studies_1 (University of Texas)	[1]	Case-Control Studies/ or Control Groups/ or Matched-Pair Analysis/ or ((case* adj5 control*) or (case adj3 comparison*) or control group*).ti,ab.	1	N/A	N/A	N/A 0
Case-control studies_2 (University of Texas)	[1]	Case-Control Studies/ or Control Groups/ or Matched-Pair Analysis/ or retrospective studies/ or ((case* adj5 control*) or (case adj3 comparison*) or control group*).ti,ab,kw.	1	N/A	N/A	N/A
Cohort studies (University of Texas)	[1]	cohort studies/ or longitudinal studies/ or follow-up studies/ or prospective studies/ or retrospective studies/ or cohort.ti,ab. or longitudinal.ti,ab. or prospective.ti,ab. or retrospective.ti,ab.	1	N/A	N/A	N/A
Clinical trials (University of Texas)	[1]	"clinical trial".pt. or "clinical trial, phase i".pt. or "clinical trial, phase ii".pt. or clinical trial, phase iii.pt. or clinical trial, phase iv.pt. or controlled clinical trial.pt. or "multicenter study".pt. or "randomized controlled trial".pt. or double-blind method/ or clinical trials as topic/ or clinical trials, phase i as topic/ or clinical trials, phase ii as topic/ or clinical trials, phase iii as topic/ or clinical trials, phase iv as topic/ or controlled clinical trials as topic/ or randomized controlled trials as topic/ or early termination of clinical trials as topic/ or multicenter studies as topic/ or ((randomi?ed adj7 trial*) or (controlled adj3 trial*) or (clinical adj2 trial*) or ((single or doubl* or tripl* or treb*) and (blind* or mask*))).ti,ab,kw. or ("4 arm" or "four arm").ti,ab,kw.	1	N/A	N/A	N/A

**Study filters (developer)**	**Source**	**Syntax according to documentation**	**Gene-ration**	**Sensi-tivity**	**Speci-ficity**	**Prec-ision**
MEDLINE Precision (Fraser 2000)	[2]	1. Comparative studies/<br>2. Follow-up studies/<br>3. (preoperat$ or pre operat$).mp<br>4. chang$.tw<br>5. evaluat$.tw<br>6. reviewed.tw<br>7. prospective$.tw<br>8. retrospective$.tw<br>9. baseline.tw<br>10. cohort.tw<br>11. consecutive$.tw<br>12. (compare$ or compara$).tw<br>13. or/1-12	3	99.5 (98.5-100)	42.9 (40.3-45.5)	20.9 (18.4-23.4)
MEDLINE Specificity (Fraser 2000)	[2]	1. Comparative studies/<br>2. Follow-up studies/<br>3. Time factors/<br>4. (preoperat$ or pre operat$).mp<br>5. chang$.tw<br>6. evaluat$.tw<br>7. reviewed.tw<br>8. prospective$.tw<br>9. retrospective$.tw<br>10. baseline.tw<br>11. cohort.tw<br>12. case series.tw<br>13. or/1-12	3	99.5 (98.5-100)	43.5 (40.4-46.6)	21.1 (18.5-23.7)

Study filters (developer)	Source	Syntax according to documentation	Gene-ration	Sensi-tivity	Speci -ficity	Prec-ision
Search terms for finding non-RCTs (Royle 2003)	[3]	A combination of the keyword terms (terms in the Title, Abstract, or Subject Heading fields) combined with the MEDLINE Publication Type (PT) terms; that is,<br>case control studies OR clinical trial* OR cohort OR comparative OR comparison OR continuing study OR control* OR cross sectional studies OR drug evaluation OR epidemiology OR follow-up studies OR intervention study OR longitudinal studies OR longterm OR matched OR phase II OR pilot projects OR prospective studies OR retrospective studies OR survival OR treatment outcome OR clinical trial (PT) OR comparative study (PT) OR multicenter study (PT)	3	85.0	N/A	N/A
Fixed method A for MEDLINE (Furlan)	[4]	Case-control studies/ or cohort studies/ or comparative study/ or cross-sectional study/ or evaluation studies/ or feasibility studies/ or follow-up studies/ or longitudinal studies/ or program evaluation/ or prospective studies/ or retrospective studies/ or treatment outcome/	3	68.6	N/A	1.5
Fixed method B for MEDLINE (Furlan)	[4]	Cohort studies/ or comparative study/ or follow-up studies/ or prospective studies/ or risk factors/ or cohort.mp. or compared.mp. or groups.mp. or multivariate.mp.	3	88.1	N/A	1.3
Medline cohort study strategy (BMJ)	[5]	1. exp cohort studies/<br>2. cohort$.tw.<br>3. controlled clinical trial.pt.<br>4. epidemiologic methods/<br>5. limit 4 to yr=1971-1988<br>6. or/1-3,5	1	N/A	N/A	N/A
Medline cohort and case-control strategy (BMJ)	[5]	1. exp cohort studies/<br>2. cohort$.tw.<br>3. controlled clinical trial.pt.<br>4. epidemiologic methods/<br>5. limit 4 to yr=1966-1989<br>6. exp case-control studies/<br>7. (case$ and control$).tw.<br>8. or/1-3,5-7	1	N/A	N/A	N/A

**Study filters (developer)**	**Source**	**Syntax according to documentation**	**Gene-ration**	**Sensi-tivity**	**Speci -ficity**	**Prec-ision**
Medline cohort, case-control, and case series strategy (BMJ)	[5]	1. exp cohort studies/<br>2. cohort$.tw.<br>3. controlled clinical trial.pt.<br>4. epidemiologic methods/<br>5. limit 4 to yr=1966-1989<br>6. exp case-control studies/<br>7. (case$ and control$).tw.<br>8. (case$ and series).tw.<br>9. or/1-3,5-8	1	N/A	N/A	N/A
Medline cohort, case-control, case series, and case study strategy (BMJ)	[5]	1. exp cohort studies/<br>2. cohort$.tw.<br>3. controlled clinical trial.pt.<br>4. epidemiologic methods/<br>5. limit 4 to yr=1966-1989<br>6. exp case-control studies/<br>7. (case$ and control$).tw.<br>8. (case$ and series).tw.<br>9 .case reports.pt.<br>10. (case$ adj2 report$).tw.<br>11. (case$ adj2 stud$).tw.<br>12. or/1-3,5-11	1	N/A	N/A	N/A

**Study filters (developer)**	**Source**	**Syntax according to documentation**	**Gene-ration**	**Sensi-tivity**	**Speci-ficity**	**Prec-ision**
Observational Studies – Medline (SIGN)	[6]	1 Epidemiologic studies/<br>2 Exp case control studies/<br>3 Exp cohort studies/<br>4 Case control.tw.<br>5 (cohort adj (study or studies)).tw.<br>6 Cohort analy$.tw.<br>7 (Follow up adj (study or studies)).tw.<br>8 (observational adj (study or studies)).tw.<br>9 Longitudinal.tw.<br>10 Retrospective.tw.<br>11 Cross sectional.tw.<br>12 Cross-sectional studies/<br>13 or/1-12	1	N/A	N/A	N/A
Therapy - Medline - Maximizes sensitivity (Haynes	[7]	clinical trial.mp. OR clinical trial.pt. OR random:.mp. OR tu.xs.	3	99.3 (98.7-99.8)	70.4 (69.8-70.9)	9.9 (9.3-10.5)
Therapy - Medline - Maximizes specificity (Haynes)	[7]	randomized controlled trial.mp. OR randomized controlled trial.pt.	3	93.1 (91.5-94.8)	97.4 (97.3-97.6)	54.4 (52.0-56.8)
Therapy - Medline - Optimising sensitivity and specificity (Haynes)	[7]	randomized controlled trial.pt. OR randomized.mp. OR placebo.mp.	3	95.8 (94.5-97.1)	95.0 (94.8-95.3)	38.5 (36.5-40.5)

**Study filters (developer)**	**Source**	**Syntax according to documentation**	**Gene-ration**	**Sensi-tivity**	**Speci -ficity**	**Prec-ision**
Cochrane Search Strategy (2008) – sensitivity-max.	[8] Prüfung: [9]	1. randomized controlled trial.pt.<br>2. controlled clinical trial.pt.<br>3. randomized.ab.<br>4. placebo.ab.<br>5. drug therapy.fs.<br>6. randomly.ab.<br>7. trial.ab.<br>8. groups.ab.<br>9. 1 or 2 or 3 or 4 or 5 or 6 or 7 or 8<br>10. exp animals/ not humans.sh.<br>11. 9 not 10	3	98.4 (97.7-99.0)	77.9 (77.5-78.3)	13.0 (12.4-13.6)
Cochrane Search Strategy (2008) – sensitivity and precision-max.	[8] Prüfung: [9]	1. randomized controlled trial.pt.<br>2. controlled clinical trial.pt.<br>3. randomized.ab.<br>4. placebo.ab.<br>5. clinical trials as topic.sh.<br>6. randomly.ab.<br>7. trial.ti.<br>8. 1 or 2 or 3 or 4 or 5 or 6 or 7<br>9. exp animals/ not humans.sh.<br>10. 8 not 9	3	97.5 (96.8-98.3)	92.4 (92.2-92.7)	30.1 (28.9-31.4)

Reference list

1. **Search filters for various databases** [http://libguides.sph.uth.tmc.edu/ovid_medline_filters]
2. Fraser C, Murray A, Burr J: **Identifying observational studies of surgical interventions in MEDLINE and EMBASE**. *BMC Med Res Methodol* 2006, **6**:41.
3. Royle P, Waugh N: **Literature searching for clinical and cost-effectiveness studies used in health technology assessment reports carried out for the National Institute for Clinical Excellence appraisal system**. *Health Technol Assess* 2003, **7**(34):iii, ix-x, 1-51.
4. Furlan AD, Irvin E, Bombardier C: **Limited search strategies were effective in finding relevant nonrandomized studies**. *J Clin Epidemiol* 2006, **59**(12):1303-1311.
5. **Study design search filters** [http://clinicalevidence.bmj.com/x/set/static/ebm/learn/665076.html]
6. **Observational studies: Medline** [http://www.sign.ac.uk/assets/search-filters-observational-studies.docx]
7. Haynes RB, McKibbon KA, Wilczynski NL, Walter SD, Werre SR: **Optimal search strategies for retrieving scientifically strong studies of treatment from Medline: analytical survey**. *BMJ* 2005, **330**(7501):1179.
8. **Searching for studies** [http://handbook.cochrane.org/chapter_6/6_searching_for_studies.htm]
9. McKibbon KA, Wilczynski NL, Haynes RB: **Retrieving randomized controlled trials from medline: a comparison of 38 published search filters**. *Health Info Libr J* 2009, **26**(3):187-202.
