# Supplementary material for: Study filters for non-randomized studies of interventions consistently lacked sensitivity upon external validation
Source: BMC Med Res Methodol. 2018 Dec 18;18:171. doi: 10.1186/s12874-018-0625-4 (PMC6299552; doi:10.1186/s12874-018-0625-4)
Supplement: Supplementary file 4 — Number of reviews and studies per Cochrane group in the reference set. This file includes information on the allocation of Cochrane reviews to the Cochrane groups (PDF 83 kb) [file 12874_2018_625_MOESM4_ESM.pdf]

Number of reviews and studies per Cochrane group in the reference set

**Cochrane group**	**Number of CRs**	**Number of studies**
Effective Practice and Organisation of Care Group	58	874
HIV/AIDS Group	26	339
Injuries Group	24	482
Gynaecological, Neuro-oncology and Orphan Cancer Group	20	321
Public Health Group	14	297
Work Group	10	205
Colorectal Cancer Group	9	183
Fertility Regulation Group	9	123
Musculoskeletal Group	8	158
Pain, Palliative and Supportive Care Group	8	116
Tobacco Addiction Group	8	192
Acute Respiratory Infections Group	7	153
Epilepsy Group	7	75
Developmental, Psychosocial and Learning Problems Group	6	111
Infectious Diseases Group	6	103
Childhood Cancer Group	5	99
Drugs and Alcohol Group	4	57
Metabolic and Endocrine Disorders Group	4	52
Stroke Group	4	82
Back and Neck Group	3	28
Consumers and Communication Group	3	17
Hepato-Biliary Group	3	85
Oral Health Group	3	28
Upper GI and Pancreatic Diseases Group	3	19
Breast Cancer Group	2	40
Movement Disorders Group	2	23
Airways Group	1	15
Anaesthesia, Critical and Emergency Care Group	1	5
Common Mental Disorders Group	1	6
Dementia and Cognitive Improvement Group	1	7
Eyes and Vision Group	1	2
Heart Group	1	16
Hypertension Group	1	20
IBD Group	1	3
Kidney and Transplant Group	1	40
Multiple Sclerosis and Rare Diseases of the CNS Group	1	3
Pregnancy and Childbirth Group	1	23
Skin Group	1	14
Urology Group	1	9
Vascular Group	1	55
Wounds Group	1	2

CR: Cochrane review; GI: Gastrointestinal; IBD: Inflammatory Bowel Disease; CNS: Central Nervous
