# Supplementary material for: Study filters for non-randomized studies of interventions consistently lacked sensitivity upon external validation
Source: BMC Med Res Methodol. 2018 Dec 18;18:171. doi: 10.1186/s12874-018-0625-4 (PMC6299552; doi:10.1186/s12874-018-0625-4)
Supplement: Supplementary file 5 — Detailed results of the evaluation of study filters. This file includes details of the filter testing (PDF 356 kb) [file 12874_2018_625_MOESM5_ESM.pdf]

Detailed results of the evaluation of study filters

The evaluation with all intervention types took place between 19.-21.06.2017. The evaluation with the subgroup of IQWiG-relevant intervention types (excluding environmental intervention, occupational intervention, health policy intervention, health systems intervention, vaccination intervention) took place between 17.-20.07.2017. Study type: 1: Randomized controlled trial (RCT), 2: Nonrandomized controlled trial, 3: Controlled before-after study, 4: Interrupted time series (with comparison group), 5: Prospective cohort study, 6: Retrospective cohort study, 7: Non-concurrent cohort study, 8: (Nested) case-control study, 9: Cross-sectional study, 10: Non-comparative study (case report or case series), 11: Before-after study, 12: Interrupted time series (without comparison group). The study types targeted by the filter are highlighted in yellow.

		All intervention types				Subgroup analysis of specific intervention			
**Study filters (developers)**	**Study type tested**	**Hits Medline***	**Test set**	**Citations found**	**Sensitivity**	**Hits Medline***	**Test set**	**Citations found**	**Sensitivity**
Case-control studies_1 (University of Texas)	1	660.864	200	56	0.28	666.144	109	23	0.21
Case-control studies_1 (University of Texas)	2	660.864	200	36	0.18	666.144	155	20	0.13
Case-control studies_1 (University of Texas)	3	660.864	200	24	0.12	666.144	100	7	0.07
Case-control studies_1 (University of Texas)	4	660.864	106	8	0.08	666.144	51	0	0.00
Case-control studies_1 (University of Texas)	5	660.864	200	19	0.10	666.144	176	18	0.10
Case-control studies_1 (University of Texas)	6	660.864	200	12	0.06	666.144	119	10	0.08
Case-control studies_1 (University of Texas)	7	660.864	31	4	0.13	666.144	10	2	0.20
Case-control studies_1 (University of Texas)	8	660.864	200	156	0.78	666.144	162	126	0.78
Case-control studies_1 (University of Texas)	9	660.864	136	7	0.05	666.144	77	6	0.08
Case-control studies_1 (University of Texas)	10	660.864	200	5	0.03	666.144	195	5	0.03
Case-control studies_1 (University of Texas)	11	660.864	200	3	0.02	666.144	147	3	0.02
Case-control studies_1 (University of Texas)	12	660.864	179	2	0.01	666.144	85	1	0.01
Case-control studies_2 (University of Texas)	1	1.284.387	200	58	0.29	1.294.799	109	24	0.22
Case-control studies_2 (University of Texas)	2	1.284.387	200	38	0.19	1.294.799	155	22	0.14
Case-control studies_2 (University of Texas)	3	1.284.387	200	30	0.15	1.294.799	100	8	0.08
Case-control studies_2 (University of Texas)	4	1.284.387	106	18	0.17	1.294.799	51	3	0.06
Case-control studies_2 (University of Texas)	5	1.284.387	200	27	0.14	1.294.799	176	26	0.15
Case-control studies_2 (University of Texas)	6	1.284.387	200	87	0.44	1.294.799	119	56	0.47
Case-control studies_2 (University of Texas)	7	1.284.387	31	12	0.39	1.294.799	10	2	0.20
Case-control studies_2 (University of Texas)	8	1.284.387	200	159	0.80	1.294.799	162	128	0.79

		All intervention types				Subgroup analysis of specific intervention			
**Study filters (developers)**	**Study type tested**	**Hits Medline***	**Test set**	**Citations found**	**Sensitivity**	**Hits Medline***	**Test set**	**Citations found**	**Sensitivity**
Case-control studies_2 (University of Texas)	9	1.284.387	136	23	0.17	1.294.799	77	11	0.14
Case-control studies_2 (University of Texas)	10	1.284.387	200	51	0.26	1.294.799	195	51	0.26
Case-control studies_2 (University of Texas)	11	1.284.387	200	11	0.06	1.294.799	147	9	0.06
Case-control studies_2 (University of Texas)	12	1.284.387	179	14	0.08	1.294.799	85	9	0.11
Cohort studies (University of Texas)	1	2.204.911	200	39	0.20	2.221.228	109	24	0.22
Cohort studies (University of Texas)	2	2.204.911	200	67	0.34	2.221.228	155	54	0.35
Cohort studies (University of Texas)	3	2.204.911	200	55	0.28	2.221.228	100	21	0.21
Cohort studies (University of Texas)	4	2.204.911	106	32	0.30	2.221.228	51	13	0.25
Cohort studies (University of Texas)	5	2.204.911	200	143	0.72	2.221.228	176	128	0.73
Cohort studies (University of Texas)	6	2.204.911	200	142	0.71	2.221.228	119	89	0.75
Cohort studies (University of Texas)	7	2.204.911	31	16	0.52	2.221.228	10	4	0.40
Cohort studies (University of Texas)	8	2.204.911	200	66	0.33	2.221.228	162	55	0.34
Cohort studies (University of Texas)	9	2.204.911	136	45	0.33	2.221.228	77	25	0.32
Cohort studies (University of Texas)	10	2.204.911	200	106	0.53	2.221.228	195	106	0.54
Cohort studies (University of Texas)	11	2.204.911	200	63	0.32	2.221.228	147	52	0.35
Cohort studies (University of Texas)	12	2.204.911	179	43	0.24	2.221.228	85	23	0.27
Clinical trials (University of Texas)	1	1.445.276	200	182	0.91	1.454.160	109	102	0.94
Clinical trials (University of Texas)	2	1.445.276	200	77	0.39	1.454.160	155	50	0.32
Clinical trials (University of Texas)	3	1.445.276	200	47	0.24	1.454.160	100	24	0.24
Clinical trials (University of Texas)	4	1.445.276	106	8	0.08	1.454.160	51	3	0.06
Clinical trials (University of Texas)	5	1.445.276	200	69	0.35	1.454.160	176	60	0.34
Clinical trials (University of Texas)	6	1.445.276	200	25	0.13	1.454.160	119	13	0.11
Clinical trials (University of Texas)	7	1.445.276	31	10	0.32	1.454.160	10	5	0.50
Clinical trials (University of Texas)	8	1.445.276	200	28	0.14	1.454.160	162	19	0.12
Clinical trials (University of Texas)	9	1.445.276	136	12	0.09	1.454.160	77	6	0.08
Clinical trials (University of Texas)	10	1.445.276	200	39	0.20	1.454.160	195	39	0.20
Clinical trials (University of Texas)	11	1.445.276	200	76	0.38	1.454.160	147	72	0.49
Clinical trials (University of Texas)	12	1.445.276	179	22	0.12	1.454.160	85	19	0.22

		All intervention types				Subgroup analysis of specific intervention			
**Study filters (developers)**	**Study type tested**	**Hits Medline***	**Test set**	**Citations found**	**Sensitivity**	**Hits Medline***	**Test set**	**Citations found**	**Sensitivity**
MEDLINE precision (Fraser 2000)	1	9.509.757	200	166	0.83	9.565.913	109	92	0.84
MEDLINE precision (Fraser 2000)	2	9.509.757	200	168	0.84	9.565.913	155	129	0.83
MEDLINE precision (Fraser 2000)	3	9.509.757	200	178	0.89	9.565.913	100	87	0.87
MEDLINE precision (Fraser 2000)	4	9.509.757	106	91	0.86	9.565.913	51	42	0.82
MEDLINE precision (Fraser 2000)	5	9.509.757	200	172	0.86	9.565.913	176	155	0.88
MEDLINE precision (Fraser 2000)	6	9.509.757	200	176	0.88	9.565.913	119	109	0.92
MEDLINE precision (Fraser 2000)	7	9.509.757	31	26	0.84	9.565.913	10	7	0.70
MEDLINE precision (Fraser 2000)	8	9.509.757	200	148	0.74	9.565.913	162	122	0.75
MEDLINE precision (Fraser 2000)	9	9.509.757	136	89	0.65	9.565.913	77	48	0.62
MEDLINE precision (Fraser 2000)	10	9.509.757	200	145	0.73	9.565.913	195	145	0.74
MEDLINE precision (Fraser 2000)	11	9.509.757	200	141	0.71	9.565.913	147	99	0.67
MEDLINE precision (Fraser 2000)	12	9.509.757	179	144	0.80	9.565.913	85	67	0.79
MEDLINE specificity (Fraser 2000)	1	8.423.107	200	153	0.77	8.471.037	109	89	0.82
MEDLINE specificity (Fraser 2000)	2	8.423.107	200	162	0.81	8.471.037	155	126	0.81
MEDLINE specificity (Fraser 2000)	3	8.423.107	200	171	0.86	8.471.037	100	88	0.88
MEDLINE specificity (Fraser 2000)	4	8.423.107	106	84	0.79	8.471.037	51	39	0.76
MEDLINE specificity (Fraser 2000)	5	8.423.107	200	168	0.84	8.471.037	176	150	0.85
MEDLINE specificity (Fraser 2000)	6	8.423.107	200	170	0.85	8.471.037	119	105	0.88
MEDLINE specificity (Fraser 2000)	7	8.423.107	31	25	0.81	8.471.037	10	8	0.80
MEDLINE specificity (Fraser 2000)	8	8.423.107	200	105	0.53	8.471.037	162	86	0.53
MEDLINE specificity (Fraser 2000)	9	8.423.107	136	75	0.55	8.471.037	77	41	0.53
MEDLINE specificity (Fraser 2000)	10	8.423.107	200	139	0.70	8.471.037	195	139	0.71
MEDLINE specificity (Fraser 2000)	11	8.423.107	200	136	0.68	8.471.037	147	97	0.66
MEDLINE specificity (Fraser 2000)	12	8.423.107	179	139	0.78	8.471.037	85	65	0.76
Search terms for finding non-RCTs (Royle	1	8.073.091	200	186	0.93	8.121.140	109	107	0.98
Search terms for finding non-RCTs (Royle	2	8.073.091	200	168	0.84	8.121.140	155	125	0.81
Search terms for finding non-RCTs (Royle	3	8.073.091	200	161	0.81	8.121.140	100	76	0.76

		All intervention types				Subgroup analysis of specific intervention			
**Study filters (developers)**	**Study type tested**	**Hits Medline***	**Test set**	**Citations found**	**Sensitivity**	**Hits Medline***	**Test set**	**Citations found**	**Sensitivity**
Search terms for finding non-RCTs (Royle	4	8.073.091	106	77	0.73	8.121.140	51	39	0.76
Search terms for finding non-RCTs (Royle	5	8.073.091	200	171	0.86	8.121.140	176	152	0.86
Search terms for finding non-RCTs (Royle	6	8.073.091	200	175	0.88	8.121.140	119	103	0.87
Search terms for finding non-RCTs (Royle	7	8.073.091	31	27	0.87	8.121.140	10	8	0.80
Search terms for finding non-RCTs (Royle	8	8.073.091	200	196	0.98	8.121.140	162	161	0.99
Search terms for finding non-RCTs (Royle	9	8.073.091	136	78	0.57	8.121.140	77	48	0.62
Search terms for finding non-RCTs (Royle	10	8.073.091	200	143	0.72	8.121.140	195	143	0.73
Search terms for finding non-RCTs (Royle	11	8.073.091	200	148	0.74	8.121.140	147	114	0.78
Search terms for finding non-RCTs (Royle	12	8.073.091	179	82	0.46	8.121.140	85	40	0.47
Fixed method A for MEDLINE (Furlan 2006)	1	4.184.894	200	103	0.52	4.212.438	109	66	0.61
Fixed method A for MEDLINE (Furlan 2006)	2	4.184.894	200	125	0.63	4.212.438	155	100	0.65
Fixed method A for MEDLINE (Furlan 2006)	3	4.184.894	200	120	0.60	4.212.438	100	59	0.59
Fixed method A for MEDLINE (Furlan 2006)	4	4.184.894	106	56	0.53	4.212.438	51	30	0.59
Fixed method A for MEDLINE (Furlan 2006)	5	4.184.894	200	147	0.74	4.212.438	176	130	0.74
Fixed method A for MEDLINE (Furlan 2006)	6	4.184.894	200	152	0.76	4.212.438	119	88	0.74
Fixed method A for MEDLINE (Furlan 2006)	7	4.184.894	31	21	0.68	4.212.438	10	6	0.60
Fixed method A for MEDLINE (Furlan 2006)	8	4.184.894	200	165	0.83	4.212.438	162	136	0.84
Fixed method A for MEDLINE (Furlan 2006)	9	4.184.894	136	67	0.49	4.212.438	77	45	0.58
Fixed method A for MEDLINE (Furlan 2006)	10	4.184.894	200	117	0.59	4.212.438	195	117	0.60
Fixed method A for MEDLINE (Furlan 2006)	11	4.184.894	200	96	0.48	4.212.438	147	63	0.43
Fixed method A for MEDLINE (Furlan 2006)	12	4.184.894	179	73	0.41	4.212.438	85	36	0.42
Fixed method B for MEDLINE (Furlan 2006)	1	6.559.073	200	144	0.72	6.596.931	109	78	0.72
Fixed method B for MEDLINE (Furlan 2006)	2	6.559.073	200	140	0.70	6.596.931	155	105	0.68
Fixed method B for MEDLINE (Furlan 2006)	3	6.559.073	200	138	0.69	6.596.931	100	66	0.66
Fixed method B for MEDLINE (Furlan 2006)	4	6.559.073	106	63	0.59	6.596.931	51	25	0.49
Fixed method B for MEDLINE (Furlan 2006)	5	6.559.073	200	170	0.85	6.596.931	176	152	0.86
Fixed method B for MEDLINE (Furlan 2006)	6	6.559.073	200	168	0.84	6.596.931	119	99	0.83
Fixed method B for MEDLINE (Furlan 2006)	7	6.559.073	31	23	0.74	6.596.931	10	6	0.60

		All intervention types				Subgroup analysis of specific intervention			
**Study filters (developers)**	**Study type tested**	**Hits Medline***	**Test set**	**Citations found**	**Sensitivity**	**Hits Medline***	**Test set**	**Citations found**	**Sensitivity**
Fixed method B for MEDLINE (Furlan 2006)	8	6.559.073	200	170	0.85	6.596.931	162	144	0.89
Fixed method B for MEDLINE (Furlan 2006)	9	6.559.073	136	94	0.69	6.596.931	77	61	0.79
Fixed method B for MEDLINE (Furlan 2006)	10	6.559.073	200	102	0.51	6.596.931	195	102	0.52
Fixed method B for MEDLINE (Furlan 2006)	11	6.559.073	200	102	0.51	6.596.931	147	66	0.45
Fixed method B for MEDLINE (Furlan 2006)	12	6.559.073	179	89	0.50	6.596.931	85	40	0.47
MEDLINE cohort study strategy (BMJ)	1	1.982.782	200	42	0.21	1.998.419	109	26	0.24
MEDLINE cohort study strategy (BMJ)	2	1.982.782	200	86	0.43	1.998.419	155	62	0.40
MEDLINE cohort study strategy (BMJ)	3	1.982.782	200	68	0.34	1.998.419	100	29	0.29
MEDLINE cohort study strategy (BMJ)	4	1.982.782	106	31	0.29	1.998.419	51	13	0.25
MEDLINE cohort study strategy (BMJ)	5	1.982.782	200	138	0.69	1.998.419	176	123	0.70
MEDLINE cohort study strategy (BMJ)	6	1.982.782	200	132	0.66	1.998.419	119	79	0.66
MEDLINE cohort study strategy (BMJ)	7	1.982.782	31	18	0.58	1.998.419	10	5	0.50
MEDLINE cohort study strategy (BMJ)	8	1.982.782	200	68	0.34	1.998.419	162	57	0.35
MEDLINE cohort study strategy (BMJ)	9	1.982.782	136	43	0.32	1.998.419	77	25	0.32
MEDLINE cohort study strategy (BMJ)	10	1.982.782	200	102	0.51	1.998.419	195	100	0.51
MEDLINE cohort study strategy (BMJ)	11	1.982.782	200	61	0.31	1.998.419	147	49	0.33
MEDLINE cohort study strategy (BMJ)	12	1.982.782	179	42	0.23	1.998.419	85	22	0.26
Medline cohort and case-control strategy	1	2.430.887	200	53	0.27	2.449.516	109	33	0.30
Medline cohort and case-control strategy	2	2.430.887	200	91	0.46	2.449.516	155	64	0.41
Medline cohort and case-control strategy	3	2.430.887	200	74	0.37	2.449.516	100	31	0.31
Medline cohort and case-control strategy	4	2.430.887	106	37	0.35	2.449.516	51	14	0.27
Medline cohort and case-control strategy	5	2.430.887	200	143	0.72	2.449.516	176	128	0.73
Medline cohort and case-control strategy	6	2.430.887	200	137	0.69	2.449.516	119	82	0.69
Medline cohort and case-control strategy	7	2.430.887	31	19	0.61	2.449.516	10	6	0.60
Medline cohort and case-control strategy	8	2.430.887	200	184	0.92	2.449.516	162	150	0.93
Medline cohort and case-control strategy	9	2.430.887	136	46	0.34	2.449.516	77	27	0.35
Medline cohort and case-control strategy	10	2.430.887	200	106	0.53	2.449.516	195	102	0.52
Medline cohort and case-control strategy	11	2.430.887	200	63	0.32	2.449.516	147	50	0.34

		All intervention types				Subgroup analysis of specific intervention			
**Study filters (developers)**	**Study type tested**	**Hits Medline***	**Test set**	**Citations found**	**Sensitivity**	**Hits Medline***	**Test set**	**Citations found**	**Sensitivity**
Medline cohort and case-control strategy (BMJ)	12	2.430.887	179	45	0.25	2.449.516	85	22	0.26
MEDLINE cohort, case-control, and case	1	2.517.309	200	54	0.27	2.536.354	109	33	0.30
MEDLINE cohort, case-control, and case	2	2.517.309	200	91	0.46	2.536.354	155	64	0.41
MEDLINE cohort, case-control, and case	3	2.517.309	200	74	0.37	2.536.354	100	31	0.31
MEDLINE cohort, case-control, and case	4	2.517.309	106	37	0.35	2.536.354	51	14	0.27
MEDLINE cohort, case-control, and case	5	2.517.309	200	143	0.72	2.536.354	176	128	0.73
MEDLINE cohort, case-control, and case	6	2.517.309	200	138	0.69	2.536.354	119	82	0.69
MEDLINE cohort, case-control, and case	7	2.517.309	31	19	0.61	2.536.354	10	6	0.60
MEDLINE cohort, case-control, and case	8	2.517.309	200	184	0.92	2.536.354	162	150	0.93
MEDLINE cohort, case-control, and case	9	2.517.309	136	47	0.35	2.536.354	77	27	0.35
MEDLINE cohort, case-control, and case	10	2.517.309	200	109	0.55	2.536.354	195	105	0.54
MEDLINE cohort, case-control, and case	11	2.517.309	200	63	0.32	2.536.354	147	50	0.34
MEDLINE cohort, case-control, and case	12	2.517.309	179	47	0.26	2.536.354	85	23	0.27
MEDLINE cohort, case-control, case series,	1	4.441.461	200	54	0.27	4.468.120	109	33	0.30
MEDLINE cohort, case-control, case series,	2	4.441.461	200	95	0.48	4.468.120	155	67	0.43
MEDLINE cohort, case-control, case series,	3	4.441.461	200	74	0.37	4.468.120	100	31	0.31
MEDLINE cohort, case-control, case series,	4	4.441.461	106	37	0.35	4.468.120	51	14	0.27
MEDLINE cohort, case-control, case series,	5	4.441.461	200	143	0.72	4.468.120	176	128	0.73
MEDLINE cohort, case-control, case series,	6	4.441.461	200	139	0.70	4.468.120	119	82	0.69
MEDLINE cohort, case-control, case series,	7	4.441.461	31	19	0.61	4.468.120	10	6	0.60
MEDLINE cohort, case-control, case series,	8	4.441.461	200	185	0.93	4.468.120	162	151	0.93
MEDLINE cohort, case-control, case series,	9	4.441.461	136	48	0.35	4.468.120	77	28	0.36
MEDLINE cohort, case-control, case series,	10	4.441.461	200	139	0.70	4.468.120	195	135	0.69
MEDLINE cohort, case-control, case series,	11	4.441.461	200	67	0.34	4.468.120	147	53	0.36
MEDLINE cohort, case-control, case series,	12	4.441.461	179	48	0.27	4.468.120	85	24	0.28
Observational Studies – Medline (SIGN)	1	2.492.125	200	37	0.19	2.510.531	109	24	0.22
Observational Studies – Medline (SIGN)	2	2.492.125	200	79	0.40	2.510.531	155	64	0.41

		All intervention types				Subgroup analysis of specific intervention			
**Study filters (developers)**	**Study type tested**	**Hits Medline***	**Test set**	**Citations found**	**Sensitivity**	**Hits Medline***	**Test set**	**Citations found**	**Sensitivity**
Observational Studies – Medline (SIGN)	3	2.492.125	200	69	0.35	2.510.531	100	30	0.30
Observational Studies – Medline (SIGN)	4	2.492.125	106	40	0.38	2.510.531	51	17	0.33
Observational Studies – Medline (SIGN)	5	2.492.125	200	143	0.72	2.510.531	176	129	0.73
Observational Studies – Medline (SIGN)	6	2.492.125	200	141	0.71	2.510.531	119	88	0.74
Observational Studies – Medline (SIGN)	7	2.492.125	31	16	0.52	2.510.531	10	5	0.50
Observational Studies – Medline (SIGN)	8	2.492.125	200	180	0.90	2.510.531	162	147	0.91
Observational Studies – Medline (SIGN)	9	2.492.125	136	67	0.49	2.510.531	77	45	0.58
Observational Studies – Medline (SIGN)	10	2.492.125	200	103	0.52	2.510.531	195	103	0.53
Observational Studies – Medline (SIGN)	11	2.492.125	200	70	0.35	2.510.531	147	46	0.31
Observational Studies – Medline (SIGN)	12	2.492.125	179	44	0.25	2.510.531	85	25	0.29
Therapy Medline (Haynes 2005) – max.	1	5.213.988	200	188	0.94	5.240.122	109	105	0.96
Therapy Medline (Haynes 2005) – max.	2	5.213.988	200	107	0.54	5.240.122	155	78	0.50
Therapy Medline (Haynes 2005) – max.	3	5.213.988	200	78	0.39	5.240.122	100	40	0.40
Therapy Medline (Haynes 2005) – max.	4	5.213.988	106	36	0.34	5.240.122	51	13	0.25
Therapy Medline (Haynes 2005) – max.	5	5.213.988	200	122	0.61	5.240.122	176	110	0.63
Therapy Medline (Haynes 2005) – max.	6	5.213.988	200	95	0.48	5.240.122	119	69	0.58
Therapy Medline (Haynes 2005) – max.	7	5.213.988	31	19	0.61	5.240.122	10	7	0.70
Therapy Medline (Haynes 2005) – max.	8	5.213.988	200	140	0.70	5.240.122	162	121	0.75
Therapy Medline (Haynes 2005) – max.	9	5.213.988	136	52	0.38	5.240.122	77	42	0.55
Therapy Medline (Haynes 2005) – max.	10	5.213.988	200	101	0.51	5.240.122	195	101	0.52
Therapy Medline (Haynes 2005) – max.	11	5.213.988	200	128	0.64	5.240.122	147	110	0.75
Therapy Medline (Haynes 2005) – max.	12	5.213.988	179	74	0.41	5.240.122	85	43	0.51
Therapy Medline (Haynes 2005) – max.	1	485.918	200	170	0.85	488.896	109	96	0.88
Therapy Medline (Haynes 2005) – max.	2	485.918	200	25	0.13	488.896	155	17	0.11
Therapy Medline (Haynes 2005) – max.	3	485.918	200	10	0.05	488.896	100	7	0.07
Therapy Medline (Haynes 2005) – max.	4	485.918	106	1	0.01	488.896	51	0	0.00
Therapy Medline (Haynes 2005) – max.	5	485.918	200	19	0.10	488.896	176	19	0.11
Therapy Medline (Haynes 2005) – max.	6	485.918	200	2	0.01	488.896	119	0	0.00

		All intervention types				Subgroup analysis of specific intervention			
**Study filters (developers)**	**Study type tested**	**Hits Medline***	**Test set**	**Citations found**	**Sensitivity**	**Hits Medline***	**Test set**	**Citations found**	**Sensitivity**
Therapy Medline (Haynes 2005) – max.	7	485.918	31	1	0.03	488.896	10	0	0.00
Therapy Medline (Haynes 2005) – max.	8	485.918	200	2	0.01	488.896	162	2	0.01
Therapy Medline (Haynes 2005) – max.	9	485.918	136	0	0.00	488.896	77	0	0.00
Therapy Medline (Haynes 2005) – max.	10	485.918	200	1	0.01	488.896	195	1	0.01
Therapy Medline (Haynes 2005) – max.	11	485.918	200	5	0.03	488.896	147	3	0.02
Therapy Medline (Haynes 2005) – max.	12	485.918	179	2	0.01	488.896	85	2	0.02
Therapy Medline (Haynes 2005) –	1	796.127	200	174	0.87	800.747	109	97	0.89
Therapy Medline (Haynes 2005) –	2	796.127	200	31	0.16	800.747	155	22	0.14
Therapy Medline (Haynes 2005) –	3	796.127	200	13	0.07	800.747	100	8	0.08
Therapy Medline (Haynes 2005) –	4	796.127	106	1	0.01	800.747	51	0	0.00
Therapy Medline (Haynes 2005) –	5	796.127	200	24	0.12	800.747	176	23	0.13
Therapy Medline (Haynes 2005) –	6	796.127	200	6	0.03	800.747	119	4	0.03
Therapy Medline (Haynes 2005) –	7	796.127	31	3	0.10	800.747	10	1	0.10
Therapy Medline (Haynes 2005) –	8	796.127	200	3	0.02	800.747	162	2	0.01
Therapy Medline (Haynes 2005) –	9	796.127	136	0	0.00	800.747	77	0	0.00
Therapy Medline (Haynes 2005) –	10	796.127	200	6	0.03	800.747	195	6	0.03
Therapy Medline (Haynes 2005) –	11	796.127	200	9	0.05	800.747	147	7	0.05
Therapy Medline (Haynes 2005) –	12	796.127	179	10	0.06	800.747	85	10	0.12
Cochrane Search Strategy (2008) –	1	3.581.596	200	185	0.93	3.601.842	109	103	0.94
Cochrane Search Strategy (2008) –	2	3.581.596	200	114	0.57	3.601.842	155	75	0.48
Cochrane Search Strategy (2008) –	3	3.581.596	200	83	0.42	3.601.842	100	42	0.42
Cochrane Search Strategy (2008) –	4	3.581.596	106	32	0.30	3.601.842	51	10	0.20
Cochrane Search Strategy (2008) –	5	3.581.596	200	105	0.53	3.601.842	176	95	0.54
Cochrane Search Strategy (2008) –	6	3.581.596	200	84	0.42	3.601.842	119	57	0.48
Cochrane Search Strategy (2008) –	7	3.581.596	31	18	0.58	3.601.842	10	6	0.60
Cochrane Search Strategy (2008) –	8	3.581.596	200	57	0.29	3.601.842	162	53	0.33
Cochrane Search Strategy (2008) –	9	3.581.596	136	27	0.20	3.601.842	77	16	0.21
Cochrane Search Strategy (2008) –	10	3.581.596	200	68	0.34	3.601.842	195	68	0.35

		All intervention types				Subgroup analysis of specific intervention			
**Study filters (developers)**	**Study type tested**	**Hits Medline***	**Test set**	**Citations found**	**Sensitivity**	**Hits Medline***	**Test set**	**Citations found**	**Sensitivity**
Cochrane Search Strategy (2008) –	11	3.581.596	200	90	0.45	3.601.842	147	75	0.51
Cochrane Search Strategy (2008) –	12	3.581.596	179	43	0.24	3.601.842	85	21	0.25
Cochrane Search Strategy (2008) –	1	1.057.717	200	181	0.91	1.064.405	109	101	0.93
Cochrane Search Strategy (2008) –	2	1.057.717	200	73	0.37	1.064.405	155	46	0.30
Cochrane Search Strategy (2008) –	3	1.057.717	200	48	0.24	1.064.405	100	26	0.26
Cochrane Search Strategy (2008) –	4	1.057.717	106	13	0.12	1.064.405	51	5	0.10
Cochrane Search Strategy (2008) –	5	1.057.717	200	32	0.16	1.064.405	176	29	0.16
Cochrane Search Strategy (2008) –	6	1.057.717	200	6	0.03	1.064.405	119	4	0.03
Cochrane Search Strategy (2008) –	7	1.057.717	31	6	0.19	1.064.405	10	3	0.30
Cochrane Search Strategy (2008) –	8	1.057.717	200	23	0.12	1.064.405	162	20	0.12
Cochrane Search Strategy (2008) –	9	1.057.717	136	6	0.04	1.064.405	77	5	0.06
Cochrane Search Strategy (2008) –	10	1.057.717	200	12	0.06	1.064.405	195	12	0.06
Cochrane Search Strategy (2008) –	11	1.057.717	200	32	0.16	1.064.405	147	27	0.18
Cochrane Search Strategy (2008) –	12	1.057.717	179	16	0.09	1.064.405	85	14	0.16
