# Supplementary material for: Study filters for non-randomized studies of interventions consistently lacked sensitivity upon external validation
Source: BMC Med Res Methodol. 2018 Dec 18;18:171. doi: 10.1186/s12874-018-0625-4 (PMC6299552; doi:10.1186/s12874-018-0625-4)
Supplement: Supplementary file 6 — Detailed results of the evaluation of study filters: only one PMID per study. This file includes a subgroup analysis of studies with only one PMID per study. (PDF 119 kb) [file 12874_2018_625_MOESM6_ESM.pdf]

Detailed results of the evaluation of study filters: only one PMID per study

The evaluation with all intervention types was performed between 19.-21.06.2017. The evaluation with the subgroup “only one PMID per study” was performed on 16.11.2017. Study types: 1: randomized controlled trial (RCT), 2: nonrandomized controlled trial, 3: controlled before-after study, 4: interrupted time series (with comparison group), 5: prospective cohort study

		All intervention types				Only 1 PMID per study			
**Filter abbreviation**	**Study type tested**	**Hits in Medline***	**Test set**	**Citations found**	**Sensitivity**	**Hits in Medline***	**Test set**	**Citations found**	**Sensitivity**
Clinical trials (University of Texas)	1	1.445.276	200	182	0.91	1.559.746	126	119	0.94
Clinical trials (University of Texas)	2	1.445.276	200	77	0.39	1.559.746	82	41	0.50
Clinical trials (University of Texas)	3	1.445.276	200	47	0.24	1.559.746	124	33	0.27
Clinical trials (University of Texas)	4	1.445.276	106	8	0.08	1.559.746	54	5	0.09
Clinical trials (University of Texas)	5	1.445.276	200	69	0.35	1.559.746	143	41	0.29
Search terms for finding non-RCTs (Royle 2003)	1	8.073.091	200	186	0.93	8.707.360	126	121	0.96
Search terms for finding non-RCTs (Royle 2003)	2	8.073.091	200	168	0.84	8.707.360	82	73	0.89
Search terms for finding non-RCTs (Royle 2003)	3	8.073.091	200	161	0.81	8.707.360	124	106	0.85
Search terms for finding non-RCTs (Royle 2003)	4	8.073.091	106	77	0.73	8.707.360	54	38	0.70
Search terms for finding non-RCTs (Royle 2003)	5	8.073.091	200	171	0.86	8.707.360	143	124	0.87
Cochrane_RCTs_Sens	1	3.581.596	200	185	0.93	3.844.364	126	120	0.95
Cochrane Search Strategy (2008) – sensitivity-max.	2	3.581.596	200	114	0.57	3.844.364	82	59	0.72
Cochrane Search Strategy (2008) – sensitivity-max.	3	3.581.596	200	83	0.42	3.844.364	124	54	0.44
Cochrane Search Strategy (2008) – sensitivity-max.	4	3.581.596	106	32	0.3	3.844.364	54	18	0.33

Cochrane Search Strategy (2008) – sensitivity-max.	5	3.581.596	200	105	0.53	3.844.364	143	72	0.50
Cochrane Search Strategy (2008) – sensitivity and precision-max.	1	1.057.717	200	181	0.91	1.135.996	126	119	0.94
Cochrane Search Strategy (2008) – sensitivity and precision-max.	2	1.057.717	200	73	0.37	1.135.996	82	42	0.51
Cochrane Search Strategy (2008) – sensitivity and precision-max.	3	1.057.717	200	48	0.24	1.135.996	124	29	0.23
Cochrane Search Strategy (2008) – sensitivity and precision-max.	4	1.057.717	106	13	0.12	1.135.996	54	6	0.11
Cochrane Search Strategy (2008) – sensitivity and precision-max.	5	1.057.717	200	32	0.16	1.135.996	143	16	0.11
